# Byssus of Green-Lipped Mussel *Perna viridis* as a Biomonitoring Biopolymer for Zinc Pollution in Coastal Waters

**DOI:** 10.3390/biology12040523

**Published:** 2023-03-30

**Authors:** Chee Kong Yap, Khalid Awadh Al-Mutairi

**Affiliations:** 1 Department of Biology, Faculty of Science, Universiti Putra Malaysia, Serdang 43400 UPM, Selangor, Malaysia; 2 Department of Biology, Faculty of Science, University of Tabuk, Tabuk P.O. Box 741, Saudi Arabia

**Keywords:** byssus, mussels, field samples, transplantation, biomonitoring

## Abstract

**Simple Summary:**

The current study aimed to confirm the use of marine mussels’ byssus (BYS) as a biomonitoring biopolymer for zinc (Zn) by comparing it to copper (Cu) and cadmium (Cd) pollution in coastal waters. The current analysis discovered four significant evidence-based points. First, the field-collected populations showed that the BYS was a more sensitive, concentrative, and accumulative biopolymer for the three metals than for total soft tissues (TST). Second, the results of the interspecific comparison showed that the mussels’ BYS was a significantly better biomonitoring biopolymer for identifying coastal areas exposed to Zn, Cd, and Cu pollution and demonstrated the role of the BYS as a route for the excretion of metal wastes. Third, the BYS was more reflective of metal bioavailability and pollution in coastal waters. Fourth, and most crucially, the field-based cage transplantation investigation amply demonstrated the accumulation and removal of the three metals by the BYS in the Straits of Johore in both contaminated and unpolluted areas. In conclusion, the mussel BYS was verified to be a superior biopolymer to TST for determining Zn (as well as Cd and Cu) bioavailability and contamination in tropical coastal waters.

**Abstract:**

The present study aimed to confirm the use of the byssus (BYS) of the green-lipped mussel *Perna viridis* as a biomonitoring biopolymer for zinc (Zn) by comparing it to copper (Cu) and cadmium (Cd) pollution in coastal waters under experimental field conditions, based on the transplantation of caged mussels between polluted and unpolluted sites in the Straits of Johore (SOJ). Four important evidential points were found in the present study. First, the 34 field-collected populations with BYS/total soft tissue (TST) ratios > 1 indicated that the BYS was a more sensitive, concentrative, and accumulative biopolymer for the three metals than TST. Significant (*p* < 0.05) and positive correlations between BYS and TST in terms of the levels of the three metals were observed. Second, the data obtained in the present study were well-supported by the interspecific comparison, which indicated that the BYS of *P. viridis* was a significantly better biomonitoring biopolymer for the identification of coastal areas exposed to Zn, Cd, and Cu pollution and played the role of an excretion route of metal wastes. Third, the higher positive correlation coefficients for the metals between the BYS sedimentary geochemical fractions than the TST sedimentary geochemical fractions indicated that the BYS was more reflective of metal bioavailability and contamination in coastal waters. Fourth, and most importantly, the field-based cage transplantation study clearly indicated the accumulation and elimination of the three metals by the BYS in both polluted and unpolluted sites in the Straits of Johore. In sum, the BYS of *P. viridis* was confirmed as a better biopolymer than TST for Zn, as well as Cd and Cu, bioavailability and contamination in tropical coastal waters.

## 1. Introduction

The main species of shallow-sea, freshwater, and deep-sea chemosynthetic environments are all members of the bivalve group known as mussels. The byssus (BYS), a proteinaceous thread used by mussels to adhere to different solid underwater surfaces, is essential to their ecology, physiology, and evolution [1]. Reinecke and Harrington [2] identified 114 marine mussels (*Mytilidae*) as sessile bivalve mollusks that live in the areas of rocky seaside habitats that are most affected by waves. The BYS, a fibrous anchor made of protein that is produced by mussels, is essential to their evolutionary survival in intertidal environments. Additionally, it maintains structural integrity under harsh chemical, mechanical, and physical conditions. Its novel usage as a matrix for water remediation has been suggested based on the combination of these qualities, which are missing in other waste materials [3]. Without exaggeration, the numerous byssal threads that make up the BYS are the organism’s lifeline. The presence of protein–metal coordination interactions in these three components is crucial, since they significantly affect the material characteristics [2]. With a strong natural coating, the cuticle of mussel byssal threads combines high extensibility with high stiffness and hardness [4].

Potentially toxic metals (PTMs) have been observed to be excreted through the BYS in *Mytilus edulis* [5,6,7] and *Perna viridis* [8,9,10]. Since there are too many metals in the cellular cytosols, and they are eventually eliminated as waste by the excretion pathway via byssal formation, the greater levels of PTMs detected in the BYS are the most likely explanation. Interestingly, the mussel BYS is a polymer network containing metals widely present in biological systems. One of the notable examples is the BYS of marine mussels, which has drawn attention for its distinctive iron-clad fiber architecture, high stability, exceptional extensibility, and self-healing qualities [11]. The potential of the BYS of P. viridis has been previously reported, but it requires further investigations [12,13].

Owing to the great usefulness and effectiveness of biomonitoring studies, there have been many reports on the use of transplanted mussels in coastal areas [14,15,16,17,18,19,20,21,22,23,24,25,26,27,28,29,30,31,32,33,34,35,36,37,38,39,40,41,42,43,44,45,46,47,48,49]. Marine mussels include *Mytilus galloprovincialis* [15,16,20,21,22,25,28,29,30,32,39], *Perna. perna* in Brazil [17], *M. edulis* [18,19,24,31,33,36,37,38], and *P. viridis* in the Gulf of Thailand [23,34]. Monitoring the metal accumulation of mussels at fixed locations is best carried out by the field-based transplantation of mussels. Marine mussels are stable and economical, making them a good choice for transplantation studies [50]. *Perna viridis*, in particular, meets the requirements, which include living a sedentary lifestyle, having enough tissue for metal analysis, feeding on suspensions, being tolerant of high heavy-metal concentrations and prone to bioaccumulating and magnifying such metals [8], and having relatively low genetic differentiation [51].

Without a field-based experimental study, any conclusions based on field-collected samples and laboratory studies would be unconvincingly justified and need more evidence. Hence, field-based experimental studies are a rational technique and are considered the most understandable and scientifically acceptable method to establish effective biomonitoring agents for chemical pollutants. This is because using transplanted mussels is an effective method for biomonitoring marine pollution [18,52,53,54,55,56,57]. Utilizing transplanted mussels reduces the effect of internal and external variables that might skew data comparisons, including seasonal fluctuations, size or age, and sexual maturity [26,32,58]. Additionally, using transplanted caged mussels is a great way to analyze heavy metals, since it minimizes ecological influences, increasing the validity of the data interpretation.

The use of *P. viridis* BYS as a biomonitoring biopolymer for cadmium (Cd), copper (Cu), and zinc (Zn) in coastal waters still requires much more experimental evidence and studies under field conditions. The above three metals are common anthropogenic PTMs in the coastal waters of Peninsular Malaysia, regardless of their essentiality differences [8,9,10]. Previously, Yap et al. [12] reported that the BYS was more sensitive and accumulative for Zn than total soft tissues (TST) according to a laboratory aquaria experimental accumulation and depuration study. Based on field-collected samples, Yap et al. [13] proposed using the BYS of *P. viridis* as a more accurate Zn biomonitoring organ according to the closer Zn correlation coefficients between the BYS and the environmental habitat sedimentary fraction than those for TST. However, a field-based experimental study on exposure to Zn-polluted waters and the elimination of Zn is still necessary. This is because a field-based experimental transplantation study of caged mussels at different sites with different metal bioavailability and contamination levels could confirm the results of studies that were merely based on field-collected and experimental aquaria experiments.

Since a further field-based experimental transplantation study was still needed to confirm our previous claim that the BYS of *P. viridis* could be used as a biopolymer monitoring material for Zn, the present study aimed to confirm the use of the *P. viridis* BYS as an effective biomonitoring biopolymer for Zn in comparison to Cd and Cu by conducting an experimental field transplantation study involving the transplantation of caged mussels between polluted and unpolluted sites in the Straits of Johore (SOJ).

## 2. Materials and Methods

### 2.1. Field-Collected Samples

Information on the field sampling of the *P. viridis* collected between 1998 and 2008 used in the present study is presented in Table S1. Before metal analysis, the mussel BYS was carefully cleaned of extraneous particulate materials [59].

### 2.2. Experimental Field-Based Transplantation of Mussel Populations

**Figure 1:**
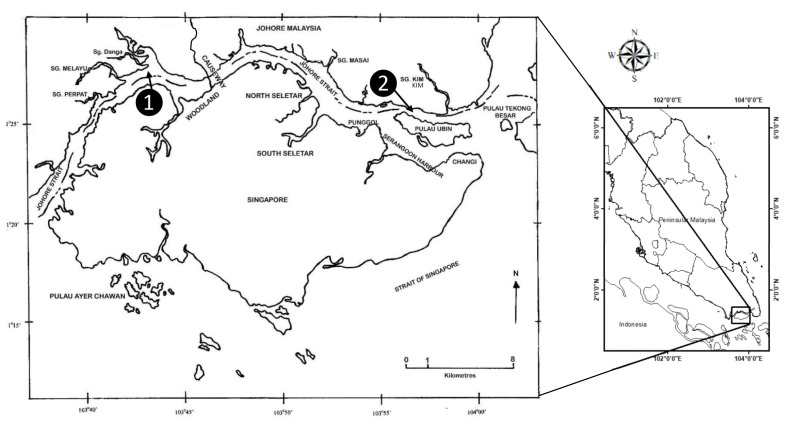
Transplantation sites of mussels at Kampung Sungai Melayu (❶) and Kampung Pasir Puteh (❷) in the Straits of Johore from the present study.

The present field-based transplantation study in the SOJ was conducted in 2009. This experimental study on accumulation and depuration was conducted on a known metal-polluted site at Kg. Pasir Puteh (KPP) and a relatively unpolluted site at Kg. Sg. Melayu (KSM) [60,61,62], both located in the SOJ (Figure 1).

About 200 *P. viridis* individuals were removed from a contaminated site at KPP and transferred to KSM in the SOJ, which is a comparatively unpolluted region. The same mussels from KSM were taken that day and transferred to KPP. The mussels were then separated into 40-specimen subgroups at random, and each subgroup was placed in a cage (20 cm × 15 cm × 18 cm) that allowed water to circulate through it. Mussels were sampled at the start of the experiment (W0), week 2 (W2), week 6 (W6), and week 10 (W10) following transplanting. For the field experiment with transplantation from KSM to KPP (M-P) and KPP to KSM (P-M) in the SOJ, the measurements of shell length, shell width, and shell height at W0, W2, W6, and W10 are shown in Table S2.

Sediment samples were taken at W0 and W6 at KPP and KSM using an Ekmen Grab. Then, these samples were placed in polyethylene bags before being transported to the lab in an ice compartment. The mussels’ soft tissues had all of their BYS entirely removed. The BYS and TST were combined from a weekly harvest of 40 mussels. The sediment, BYS, and TST samples were dried at 105 °C to a constant dry weight [63]. All the dried samples of BYS and TST were digested in concentrated nitric acid (AnalaR grade, BDH 69%). The dried sediment samples were all sieved through a 63 μm stainless steel aperture and vigorously agitated to establish homogeneity. The sieved sediments were digested in a mixture of concentrated nitric acid (AnalaR grade, BDH 69%) and perchloric acid (60%) (ratio 4:1). They were first placed in a hot-block digestor at a low temperature (40°) for one hour before increasing the temperature to 140 °C for three hours. The digested samples were diluted with double-distilled water to reach a 40 mL volume. The filtrate was stored until metal analysis in an acid-washed pillbox after filtering by using Whatman No. 1 filter paper.

Additionally, the geochemical fractions of Cd, Cu, and Zn in the sediment samples were measured using Yap et al. [63] and Badri and Aston [64]’s modified sequential extraction technique (SET). They were classified as “easily, readily leachable or exchangeable” (F1), “acid-reducible” (F2), “oxidizable organic” (F3), and “resistant” (F4).

### 2.3. Metal Identification

A Perkin-Elmer Model AAnalyst 800 air-acetylene flame atomic absorption spectrophotometer was used to analyze the samples for Cd, Cu, and Zn after filtering (AAS). The data are presented in mg/kg dry weight. The accuracy of the analysis was confirmed using blanks, and all of the glassware and equipment used were acid-washed to prevent contamination. As shown in Table S3, Certified Reference Materials were employed to verify the results, i.e., samples of dogfish liver (DOLT-3, National Research Council Canada) and Chinese soil (NSC DC73319) (CRM).

### 2.4. Statistical Analysis

Using Kaleida Graphs, version 5.0, the given data’s overall statistics and graphical histograms were produced (1986–2022 by Synergy Software, Eden Prairie, MN, USA). As the most popular approach for identifying non-normality when the sample size is modest (N < 50), the Shapiro–Wilk test was used [65,66,67]. These data, which significantly varied from a normal distribution (based on the Shapiro–Wilk normality test; 0.05), were transformed using the log_10_ (value + 1) method before correlation analysis and multiple linear (forward) stepwise regression analysis (MLSRA). In Table S4, the results of a normality test using the Shapiro–Wilk method on the metal data from the 34 populations considered in the correlation and regression analysis are shown. This log_10_ transformation was employed to stabilize the variance and the lack of normality in order to construct a frequency distribution that was more comparable to a normal distribution and to meet the need of normality for the three statistical analytical models [68,69]. STATISTICA was used to carry out the MLSRA and correlation analysis (Version 10; StatSoft. Inc., Tulsa, OK, USA, 1984–2011). Studies on the links between a dependent variable and independent factors have demonstrated this in several instances [70,71,72,73,74,75].

The sedimentary geochemical fractions (F1, F1, F3, F4, and SUM) were the independent factors for the MLSRA, whereas the dependent variables were the BYS and TST of 34 populations of field-collected mussels.

## 3. Results

### 3.1. Mussel Transplantation in the Straits of Johore

According to Table S5, the concentrations of Cd, Cu, and Zn in the sediments and their geochemical fractions collected from KPP in this investigation were generally greater than those from KSM. They agreed with those reported by [60,61,62] from the same sample site. In light of this, it was shown that KPP had greater amounts of metal contamination than KSM. In the SOJ, there are large shipyard repair and construction facilities, fossil-fuel-fired electrical power plants, shipping dock activities, and nearby shipyards that use biofouling paints, which were the likely causes for the high availability of metals at KPP [76,77,78,79]. However, the only operations present at KSM are mussel and fish aquaculture, and the area is said to have little human activity [60,61,62].

**Figure 2:**
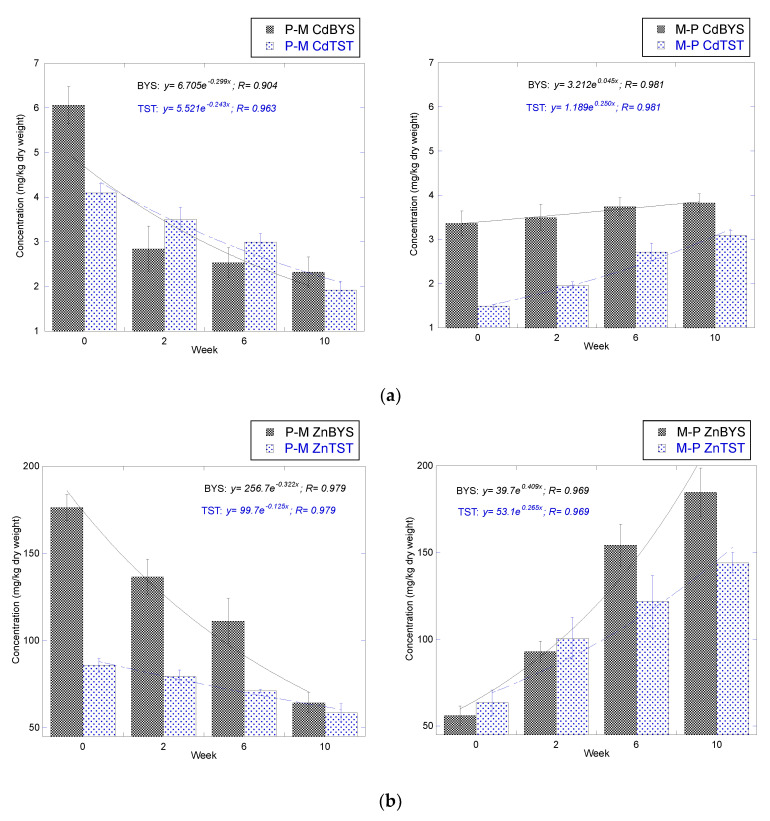

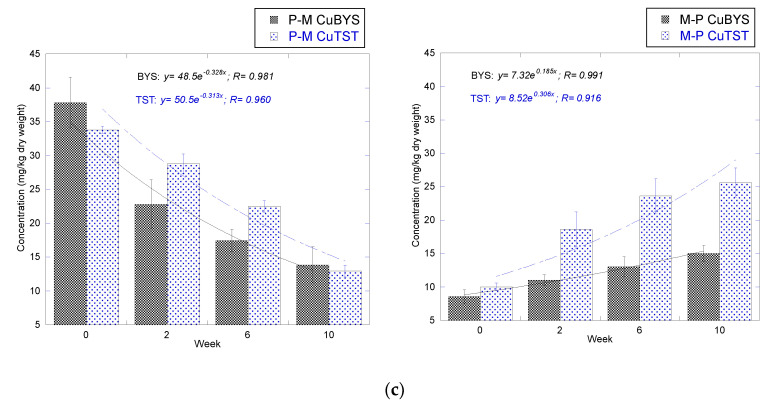
Accumulation and depuration of (**a**) Cd, (**b**) Zn, and (**c**) Cu concentrations (mg/kg dry weight) in the byssus (BYS) and total soft tissues (TST) of *Perna viridis* transplanted from Kg. Pasir Puteh to Kg. Sungai Melayu (P-M; **left**), and from Kg. Sungai Melayu to Kg. Pasir Puteh (M-P; **right**). Curve fits were based on exponential equations.

The accumulation and depuration of Cd, Cu, and Zn concentrations in the BYS and TST of *P. viridis* transplanted from KPP to KSM and from KSM to KPP are presented in Figure 2.

#### 3.1.1. Cd

For Cd in the KPP to KSM group (Figure 2a), a slightly faster depuration constant (decay of −0.299) in the BYS than in the TST (−0.243) could be observed. The higher (but not significant) levels of Cd in the TST compared to the BYS after 2, 6, and 10 weeks of depuration showed that Cd could be tightly bound to metallothionein in the TST, resulting in lower Cd levels in the BYS, which acted as an excretion route of Cd. After 10 weeks of depuration at KSM, the Cd level in the TST was 1.92 mg/kg, which was still higher (28.9% higher) than the mussels initially collected from KSM (1.49 mg/kg). This could have been due to the fact that 28.9% of the accumulated Cd was detoxified inside the tissues, as it would have been almost all tightly bound to MT during storage. For the BYS, this figure reached 2.32 mg/kg, which was 31.0% lower than that (3.36 mg/kg) of the local mussels at KSM.

In contrast, for Cd in the KSM to KPP group (Figure 2a), a faster accumulation constant (a constant of 0.250) in the TST than in the BYS (0.045) was obtained. It was clearly seen that the Cd levels in the BYS were significantly higher than in the TST during the accumulation periods of weeks 0, 2, 6, and 10. This showed that the increment in Cd levels in the TST also caused an increment in the Cd level in the BYS, but at a slower rate. This also meant that the accumulation of Cd in the BYS was significantly higher than in the TST. The lower Cd accumulation in the TST could be excreted via the BYS, causing elevated Cd levels in the BYS. After 10 weeks of accumulation, the amount in the TST reached 3.08 mg/kg, which was 24.7% lower than that (4.09 mg/kg) of the initial KPP mussels’ TST. For the BYS, it reached 3.81 mg/kg, which was 37.1% lower than that (6.06 mg/kg) of the initial KPP mussels’ BYS.

#### 3.1.2. Zn

For Zn in the KPP to KSM group (Figure 2b), a faster depuration constant (decay of −0.322, compared to −0.299 for Cd) in the BYS than in the TST (−0.125, compared to −0.243 for Cd) could be observed. The significantly (*p* < 0.05) higher levels of Zn in the TST than in the BYS after 2 and 6 weeks of depuration showed that Zn could be highly or partially regulated and less bound to metallothionein in the TST, resulting in high Zn levels in the BYS, which acted as an excretion route of Zn. After 10 weeks of depuration at KSM, the Zn level in the TST was 58.7 mg/kg, almost reaching the Zn levels (only 7.63% lower) of the local mussels’ TST (63.5 mg/kg). For the BYS, it reached 64.4 mg/kg, which was 15.0% higher than that (56.0 mg/kg) of the local mussels at KSM. This meant that *P. viridis* was capable of depurating the Zn from the local mussels at KSM. It was also assumed that the Zn was partially regulated and not tightly bound to MT (detoxified storage), as shown by the significantly higher Zn levels in the BYS, especially after weeks 2 and 6 of depuration.

Similarly, For Zn in the KSM to KPP group (Figure 2b), a faster accumulation constant (0.409, compared to 0.045 for Cd) in the BYS than in the TST (0.265, compared to 0.250 for Cd) was obtained. Interestingly, it was clearly seen that the Zn levels in the BYS were significantly higher than in the TST during the accumulation periods of weeks 6 and 10 (similar to Cd accumulation). This showed that the increment in Zn levels in the TST also caused an increment in the Cd level in the BYS but at a slower rate, especially at weeks 6 and 10. Similarly to Cd, this also meant that the accumulation of Zn in the BYS was significantly higher than in the TST. This indicated that Zn was partially regulated via the BYS, and the BYS acted as an excretion route of Zn, since higher Zn levels were found in the BYS than in the TST. After 10 weeks of accumulation, the amount in the TST reached 144 mg/kg, which was 68.2% higher than that (85.8 mg/kg) of the initial KPP mussels’ TST. For the BYS, it reached 185 mg/kg, which was 4.82% higher than that (176 mg/kg) of the initial KPP mussels’ BYS.

#### 3.1.3. Cu

For Cu in the KPP to KSM group (Figure 2c), a slightly faster depuration constant (decay of −0.328, compared to −0.322 for Zn and −0.299 for Cd) in the BYS than in the TST (−0.313, compared to −0.125 for Zn and −0.243 for Cd) could be observed. Interestingly, significantly higher levels of Cu in the TST than the BYS were observed at weeks 2 and 6 (not found in the Zn study), indicating that the Cu levels were a function of the assimilation of the TST of *P. viridis* and that Cu could be less partially regulated and more tightly bound to metallothionein in the TST, resulting in higher Zn levels in the TST than the BYS. Similar patterns of a decrement in Cu levels between the BYS and TST (with higher Cu levels in the BYS at weeks 2 and 6) also well-supported the above assumption. After 10 weeks of depuration at KSM, the Cu level in the TST was 12.9 mg/kg, which was still higher (29.2% higher) than the Cu levels (10.0 mg/kg) of the local mussels’ TST at KSM. For the BYS, it reached 13.9 mg/kg, which was 62.0% higher than that (8.55 mg/kg) of the local mussels at KSM. This meant that *P. viridis* could depurate the Cu but not reach the local mussels’ Cu levels. This indicated that the Cu was slightly tightly bound to MT (detoxified storage) and harder to depurate at week 10. This result further supported the partial regulation of Zn.

Similarly, for Cu in the KSM to KPP group (Figure 2c), a lower accumulation constant (0.185, compared to 0.409 for Zn and 0.045 for Cd) in the BYS than in the TST (0.306, compared to 0.265 for Zn and 0.250 for Cd) was also obtained. Interestingly, it was clearly seen that the Cu levels in the TST were significantly higher than in the BYS during the accumulation periods of weeks 2, 6, and 10 (in contrast to Zn accumulation, in which the BYS was higher than the TST). This showed that the increment in Cu levels in the TST also caused an increment in Cu level in the BYS but at a slower rate. In contrast to Cd and Zn, this meant that the accumulation of Cu in the TST was significantly higher than in the BYS. This indicated that Cu was not partially regulated, as found for Zn, via the BYS, since higher Cu levels were found in the TST than in the BYS. After 10 weeks of accumulation, the amount in the TST reached 25.6 mg/kg, which was 24.2% lower than that (33.8 mg/kg) of the initial KPP mussels’ TST. For the BYS, it reached 15.0 mg/kg, which was 60.3% lower than that (37.8 mg/kg) of the initial KPP mussels’ BYS.

### 3.2. Ratios of Metals between Byssus and Total Soft Tissues

#### 3.2.1. Cd

The overall statistics of the concentrations of Cd in the BYS and TST of *P. viridis* and their ratios (BYS/TST) collected from 34 populations in the coastal waters of Peninsular Malaysia (PM) and two sites in the SOJ during the experimental study, along with those reported in the literature, are presented in Table 1 and Table S6.

The Cd concentrations in the TST for the experimental transplantation study in the SOJ ranged from 1.49 to 4.09 mg/kg dry weight, compared to the wider Cd range (2.32–6.06) in the BYS. The ratios of CdBYS/CdTST ranged from 0.81 to 2.25, with a mean of 1.38. The Cd concentrations in the TST of the 34 populations ranged from 0.35 to 7.04 mg/kg dry weight, compared to the narrower Cd range (0.48–6.06 mg/kg dry weight) in the BYS. The ratios of CdBYS/CdTST ranged from 0.07 to 4.55, with a mean of 1.81 (Table 1).

The cited Cd data in the BYS and TST of different *Mytilidae* families are presented in Table S7. The Cd concentrations in the TST of *P. viridis* from KotO and Kennedy, Hong Kong [80] ranged from 1.02 to 5.40 mg/kg dry weight, compared to the narrower Cd range (0.77–1.83 mg/kg dry weight) in the BYS. The ratios of CdBYS/CdTST ranged from 0.28 to 1.16, with a mean of 0.60. The Cd concentrations in the TST of *M. edulis* from Masan and Ulsan, Korea [81] ranged from 0.63 to 9.98 mg/kg dry weight, compared to the narrower Cd range (0.19–5.40 mg/kg dry weight) in the BYS. The ratios of CdBYS/CdTST ranged from 0.23 to 1.89, with a mean of 0.71.

The Cd concentrations in the TST of *M. edulis trossulus* from the Pomeranian Bay of the Baltic Sea [59] ranged from 1.74 to 6.07 mg/kg dry weight, compared to the narrower Cd range (0.28–3.25 mg/kg dry weight) in the BYS. The ratios of CdBYS/CdTST ranged from 0.06 to 0.74, with a mean of 0.32. The Cd concentrations in the TST of *M. edulis trossulus* from the Supsk Bank of the Baltic Sea [59] ranged from 2.71 to 5.57 mg/kg dry weight, compared to the narrower Cd range (0.62–3.65 mg/kg dry weight) in the BYS. The ratios of CdBYS/CdTST ranged from 0.20 to 1.07, with a mean of 0.55.

The Cd concentrations in the TST of *M. edulis trossulus* from the Gulf of Gdansk in the Baltic Sea [59] ranged from 1.23 to 3.12 mg/kg dry weight, compared to the narrower Cd range (0.30–1.20 mg/kg dry weight) in the BYS. The ratios of CdBYS/CdTST ranged from 0.19 to 0.63, with a mean of 0.42. The Cd concentrations in the TST of *M. edulis* from Japan [6,82] ranged from 0.92 to 18.4 mg/kg dry weight, compared to the narrower Cd range (0.16–1.05 mg/kg dry weight) in the BYS. The ratios of CdBYS/CdTST ranged from 0.03 to 0.88, with a mean of 0.36.

#### 3.2.2. Zn

The overall statistics of the concentrations of Zn in the BYS and TST of *P. viridis* and their ratios (BYS/TST) collected from 34 populations in the coastal waters of Peninsular Malaysia (PM) and two sites in the SOJ during the experimental study, along with those reported in the literature, are presented in Table 2 and Table S8.

The Zn concentrations in the TST from the experimental transplantation study in the SOJ ranged from 58.7 to 144 mg/kg dry weight, compared to the wider Zn range (56.0–185) in the BYS. The ratios of ZnBYS/ZnTST ranged from 0.88 to 2.05, with a mean of 1.35. The Zn concentrations in the TST of the 34 populations ranged from 50.9 to 130 mg/kg dry weight, compared to the wider Zn range (34.9–256) in the BYS. The ratios of ZnBYS/ZnTST ranged from 0.36 to 2.91, with a mean of 1.35 (Table 2).

The cited Zn data in the BYS and TST of different *Mytilidae* families are presented in Table S7. The Zn concentrations in the TST of *P. viridis* from KotO and Kennedy, Hong Kong [80] ranged from 104 to 152 mg/kg dry weight, compared to the wider Zn range (103–342 mg/kg dry weight) in the BYS. The ratios of ZnBYS/ZnTST ranged from 0.90 to 2.71, with a mean of 1.65. The Zn concentrations in the TST of *M. edulis* from Masan and Ulsan, Korea [81] ranged from 107 to 279 mg/kg dry weight, compared to the wider Zn range (81.1–536 mg/kg dry weight) in the BYS. The ratios of ZnBYS/ZnTST ranged from 0.68 to 3.00, with a mean of 2.01.

The Zn concentrations in the TST of *M. edulis trossulus* from the Pomeranian Bay of the Baltic Sea [59] ranged from 102 to 193 mg/kg dry weight, compared to the wider Zn range (87.9–396 mg/kg dry weight) in the BYS. The ratios of ZnBYS/ZnTST ranged from 0.62 to 2.37, with a mean of 1.40. The Zn concentrations in the TST of *M. edulis trossulus* from the Supsk Bank of the Baltic Sea [59] ranged from 124 to 157 mg/kg dry weight, compared to the wider Zn range (120–264 mg/kg dry weight) in the BYS. The ratios of ZnBYS/ZnTST ranged from 0.88 to 2.12, with a mean of 1.23.

The Zn concentrations in the TST of *M. edulis trossulus* from the Gulf of Gdansk in the Baltic Sea [59] ranged from 100 to 151 mg/kg dry weight, compared to the wider Zn range (121–226 mg/kg dry weight) in the BYS. The ratios of ZnBYS/ZnTST ranged from 0.94 to 1.77, with a mean of 1.30. The Zn concentrations in the TST of *M. edulis* from Japan [6,82] ranged from 127 to 360 mg/kg dry weight, compared to the wider Zn range (98.4–297 mg/kg dry weight) in the BYS. The ratios of ZnBYS/ZnTST ranged from 0.77 to 1.00, with a mean of 0.87.

#### 3.2.3. Cu

The overall statistics of the concentrations of Cu in the BYS and TST of *P. viridis* and their ratios (BYS/TST) collected from 34 populations in the coastal waters of Peninsular Malaysia (PM) and two sites in the SOJ during the experimental study, along with those reported in the literature, are presented in Table 3 and Table S9.

The Cu concentrations in the TST from the experimental transplantation study in the SOJ ranged from 10.0 to 33.8 mg/kg dry weight, compared to the wider Cu range (8.55–37.8 mg/kg dry weight) in the BYS. The ratios of CuBYS/CuTST ranged from 0.55 to 1.23, with a mean of 0.79. The Cu concentrations in the TST of the 34 populations ranged from 2.82 to 20.1 mg/kg dry weight, compared to the wider Cu range (8.55–135 mg/kg dry weight) in the BYS. The ratios of CuBYS/CuTST ranged from 0.83 to 6.26, with a mean of 3.26 (Table 3).

The cited Cu data in the BYS and TST of different *Mytilidae* families are presented in Table S7. The Cu concentrations in the TST of *P. viridis* from KotO and Kennedy, Hong Kong [80] ranged from 10.1 to 18.0 mg/kg dry weight, compared to the wider Cu range (32.6–111 mg/kg dry weight) in the BYS. The ratios of CuBYS/CuTST ranged from 2.38 to 6.17, with a mean of 3.98. The Cu concentrations in the TST of *M. edulis* from Masan and Ulsan, Korea [81] ranged from 6.99 to 58.8 mg/kg dry weight, compared to the wider Cu range (21.9–211) in the BYS. The ratios of CuBYS/CuTST ranged from 2.74 to 6.31, with a mean of 4.51.

The Cu concentrations in the TST of *M. edulis trossulus* from the Pomeranian Bay of the Baltic Sea [59] ranged from 6.63 to 23.9 mg/kg dry weight, compared to the wider Cu range (17.9–58.1 mg/kg dry weight) in the BYS. The ratios of CuBYS/CuTST ranged from 2.37 to 2.96, with a mean of 2.61. The Cu concentrations in the TST of *M. edulis trossulus* from the Supsk Bank of the Baltic Sea [59] ranged from 7.62 to 10.3 mg/kg dry weight, compared to the wider Cu range (11.0–32.6 mg/kg dry weight) in the BYS. The ratios of CuBYS/CuTST ranged from 1.40 to 3.17, with a mean of 2.50.

The Cu concentrations in the TST of *M. edulis trossulus* from the Gulf of Gdansk in the Baltic Sea [59] ranged from 5.70 to 8.93 mg/kg dry weight, compared to the wider Cu range (17.3–33.6 mg/kg dry weight) in the BYS. The ratios of CuBYS/CuTST ranged from 2.40 to 4.56, with a mean of 3.45. The Cu concentrations in the TST of *M. edulis* from Japan [6,82] ranged from 3.76 to 385 mg/kg dry weight, compared to the wider Cu range (18.8.4–1870) in the BYS. The ratios of CuBYS/CuTST ranged from 3.52 to 16.9, with a mean of 7.57.

### 3.3. Correlation of Metals between Byssus and Habitat Sedimentary Geochemical Fractions

The correlation coefficients of the metals (Cd, Cu, and Zn) between the BYS and TST of the 34 *P. viridis* populations and their geochemical fractions in the surface sediments are presented in Table 4. It was shown in the pairwise comparison between ZnBYS and ZnTST that they were significantly (*p* < 0.05) and positively correlated (R = 0.39). Similarly, the pairwise comparison between CuBYS and CuTST also showed significant (*p* < 0.05) and positive correlation (R = 0.69). However, the pairwise comparison between CdBYS and CdTST showed weak and insignificant correlation (R = −0.16; *p* > 0.05). This indicated that the BYS could be an excretion route for Zn and Cu but not for Cd. Therefore, the BYS is an excretion route for Zn and Cu but not for metals in general.

Interestingly, ZnBYS correlated positively with CuBYS (R = 0.66; *p* < 0.05) and CuTST (R = 0.36; *p* < 0.05). This could be related to the fact that Cu and Zn are essential metals for mussels. However, it was also clearly shown that ZnBYS negatively correlated with CdTST (R = −0.43; *p* < 0.05) and weakly correlated with CdBYS (R = 0.13; *p* > 0.05). This indicated that Cd is not an essential metal.

Higher correlation coefficients were found for pairwise comparisons between ZnBYS and ZnF2, ZnBYS and ZnF3, and ZnBYS and ZnSUM than for similar pairwise comparisons between ZnTST and ZnF2, ZnTST and ZnF3, and ZnTST and ZnSUM, but they were not significant (*p* > 0.05). Higher significant (*p* < 0.05) correlation coefficients were found for pairwise comparisons between CuBYS and CuF3, CuBYS and CuF4, and CuBYS and CuSUM than for similar pairwise comparisons between CuTST and CuF3, CuTST and CuF4, and CuTST and CuSUM. Higher correlation coefficients were found for pairwise comparisons between CdBYS and CdF1, CdBYS and CdF2, and CdBYS and CdF3 than for similar pairwise comparisons between CdTST and CdF1, CdTST and CuF2, and CdTST and CdF3, but they were not significant (*p* > 0.05).

### 3.4. Multiple Linear Stepwise Regression Analytical Outputs Based on BYS and TST as Dependent Variables

The multiple linear stepwise regression analytical outputs based on the BYS and TST of *P. viridis* (as dependent variables) and the geochemical fractions of the three metals (as independent variables) collected from 34 populations in the coastal waters of Peninsular Malaysia are presented in Table 5. For Zn, eight independent variables (including ZnTST) were selected as influential factors for the accumulation of ZnBYS. Even though eight independent variables (excluding ZnBYS) were also selected as influential factors for ZnTST, only three similar parameters (CuF2, CdF3, and CuF1) were selected as influential factors for ZnBYS and ZnTST. This indicated that the processes of the accumulation and regulation of Zn differed based on the (5/8) variables selected as influential factors for the accumulation of ZnBYS and ZnTST.

For Cu, six independent variables (including CuTST) were selected as influential factors for the accumulation of CuBYS. Even though six independent variables (including CuBYS) were also selected as influential factors for CuTST, only one similar parameter (ZnTST) was selected as an influential factor for both CuBYS and CuTST. This indicated that the processes of the accumulation and regulation of Cu differed based on the (5/6) variables selected as influential factors for the accumulation of CuBYS and CuTST.

For Cd, four independent variables (including CdTST) were selected as influential factors for the accumulation of CdBYS. However, ten independent variables (including CdBYS) were selected as influential factors for the accumulation of CdTST. Only one similar parameter (CuF2) was selected as an influential factor for both CdBYS and CdTST. This indicated that the processes of the accumulation and regulation of Cd differed based on the variables selected as influential factors for the accumulation of CdBYS and CdTST.

## 4. Discussion

### 4.1. Higher Metal Accumulation in the Byssus Than in the Soft Tissues: A Sensitive Organ

The 34 field-collected populations of *P. viridis* with higher levels of metals in the BYS than the TST indirectly implied that the mussel BYS biopolymer is more sensitive to the metal bioavailability of polluted waters. The experimental accumulation study of *P. viridis* in the SOJ confirmed the higher levels of metals in the BYS than in the TST.

The identical patterns of metal accumulation and elimination in the BYS and TST of *P. viridis* revealed that metabolic processes, rather than direct contact with the surrounding seawater, were primarily responsible for the metal levels in both tissues. According to Ikuta [6,7] and Yap et al. [8], metal level regulation in the TST causes heavy metals to be transported metabolically to the BYS when the metal levels are high. According to Phillips and Rainbow [83] and Phillips [84], regulation and sequestration are crucial strategies for reducing the negative effects of excessive amounts of these metals. This may be the reason why *P. viridis*’s BYS metal levels were higher than the TST metal levels.

The reported research’s interspecific comparisons provide strong support for the increased metal accumulation in the BYS compared to the TST. The results for the BYS of *M. edulis* and *P. viridis* published by Ikuta [6,7], Szefer et al. [82], and Yap et al. [8] were comparable to these findings. The validity of our strategy of using interspecific mussel comparisons is further supported by the intercomparison studies of Blackmore and Wang [85] carried out at various local and global scales, which revealed little variation between four species of mytilids (*M. edulis*, *M. galloprovincialis*, *M. trossulus*, and *P. viridis*) in the parameters influencing the bioaccumulation of Zn and Cd. Their findings strongly encouraged the inclusion of multiple mussel species in international mussel watch programs, since they demonstrated that concentration data from different temperature zones, even when using different mytilid species, may be directly comparable.

Szefer et al. (2002) discovered that the BYS of *M. edulis trossulus* successfully concentrated Cd while only weakly concentrating Hg and Zn at 23 sites along the Polish Baltic Sea coast. In contrast to the TST, the BYS concentrated Pb; Cu; Cr; and, particularly, Ag, Ni, Mn, and Fe to a greater extent. Szefer et al. [59] found the *M. edulis* BYS to be a significantly better bioindicator for detecting coastal regions exposed to metallic pollution. The mussel BYS is a sensitive monitor of Pb, Cu, Co, Cr, Ni, Fe, and Mn, according to Szefer et al. [81], because it collects more metal than the TST [82,86]. The data gathered in this study for byssal Cu, Pb, Co, Ni, Fe, and (partly) Zn in severely contaminated sample locations of Ulsan Bay were comparable to those reported elsewhere, such as the Saganoseki region and Kyushu Island [82,86].

The higher concentrations of Cd, Zn, and Cd in the BYS samples from both polluted and uncontaminated populations revealed that this organ might serve as a metal excretion channel. This phenomenon was brought on by the mussel BYS, which has been well-characterized in *Mytilus* species [59,82] and in *P. viridis* for Cd, Pb, and Zn and is employed as an excretion route for Cd, Zn, and Cu [8,13].

### 4.2. Higher Correlation Coefficients of Metals with Environmental Sedimentary Fractions in Byssus Than Total Soft Tissues

The present study further confirmed the use of the *P. viridis* BYS as a biopolymer monitoring organ for Zn pollution. Yap et al. [12] supported this further by first suggesting that the BYS of *P. viridis* could be a biopolymer monitoring organ for Zn. Later, Yap et al. [13] discovered that there was a significant (*p* < 0.01) Pearson’s correlation coefficient (R = 0.84) between the Zn concentrations in the BYS and TST, indicating that the BYS may function as a conduit for Zn excretion. This demonstrated that the BYS was more accurate than the TST in capturing Zn contamination in the field.

Szefer et al. [59] found a significant link between the Cd concentrations in the TST and seawater (*p* < 0.05). The higher levels of Cu and Zn and lower levels of Cd in the BYS may be explained by the much more efficient transfer of Cu and Zn from the TST to the BYS. The hepatopancreas heavily accumulates Cd in the mussel TST [87]. This might be one of the reasons why the BYS had higher concentrations of Cu and Zn than Cd, which mostly accumulated in the TST and was found in areas heavily polluted by hazardous metals.

Many such relationships between biomonitors and environmental sediments in terms of metals have been reported to prove biomonitors’ capabilities for detecting specified metal(s), such as the studies by Yap et al. [63] on the TST of *P. viridis* for Cd, Cu, and Zn; Yap et al. [88] on shells for Zn; Yap et al. [13] on the BYS for Zn; and Shulkin et al. [35] regarding metal concentrations in the TST of *Mytilus grayanus* and oyster *Crassostrea gigas*.

### 4.3. Byssus as an Effective Biopolymer for the Monitoring of Zn Pollution

Besides having higher correlation coefficients for Zn with the environmental sedimentary fractions than the TST, the BYS was proven to be an effective biopolymer for monitoring Zn pollution in coastal waters.

The depuration of Zn in the TST from KPP was as much as 7.63% lower than that at KSM, but the Cd and Cu levels in the TST were nearly 30% higher than those at KSM. The metal levels in the BYS were even higher than in the TST, that is, the Zn levels in the BYS from KPP were lower (15%) than those at KSM, while the Cd and Cu levels in the BYS were higher (62.0% for Cu; 31% for Cd) than those at KSM. This clearly showed the different elimination patterns between Zn and Cd (and Cu) in the BYS and TST. This strongly indicated that the Cd and Cu in the TST were more tightly bound to MT when compared to Zn. The Zn in the TST could be more easily mobilized; therefore, Zn was easily depurated through the BYS at even lower rates than Cd and Cu at KSM after 10 weeks of depuration. Due to the mussels’ readily mobilized compartments (defecation, surface absorption, mucus, and low-affinity ligands), the Zn was quickly released during the depuration phase [89]. The quick loss also suggested that Zn excretion may happen via the BYS [90]. This implied that the BYS could act as an effective biopolymer for monitoring Zn pollution during elimination in unpolluted coastal waters.

Some bivalve transplantation studies documented in the literature, such as those reported by Gabr and Gab-Alla [91] for clams (*Ruditapes decussatus* and *Venerupis pullastra*) transplanted from a polluted site to a clean site, support the anticipated lower levels of metals in the TST of polluted bivalves transplanted to a clean site. Hedouin et al. [26] also noted lower concentrations of Cr and Cu in *Isognomon isognomon* and Co, Ni, and Zn in *Gafrarium tumidum* transplanted from a contaminated location to a clean site in a New Caledonia lagoon.

The values for Cu (62.0%) and Cd (31%) that were still higher than those of the local population in the TST of *P. viridis* at KSM after 10 weeks of depuration could have been due to several factors. The levels of Cd and Cu in the TST of *P. viridis* were much lower than those observed in the initial population at KSM after 10 weeks of depuration, which might have resulted from the accumulation being reliant on the transplanting time [26]. This demonstrated that over the 10 weeks of transplanted *P. viridis* depuration, the metals were not completely eliminated. The equilibration in trace metals between bivalves and their environments might take anywhere from 30 days to 77 days, even longer for some species, according to previous research [44,45,92,93]. Because of past exposure histories, the diverse life phases of bivalves, metabolic activities linked to adaptation to a new habitat, temperature variations, and food availability, there may be vast variation in equilibration timeframes among species [94].

The accumulation of Zn in the TST from KSM was 68.2% higher than that at KPP, but the Cd and Cu levels did not reach those at KPP (the accumulation of both metals was about 25% lower than that at KPP). However, the metal levels in the BYS were even lower than in the TST, that is, the Zn levels in the BYS from KSM were slightly higher (4.82%) than those at KPP, while the Cd and Cu levels in the BYS were significantly lower (Cu: 60.3%; Cd: 37.1%) than those at KPP. This clearly showed the different accumulation patterns between Zn and Cd (and Cu) in the BYS and TST. This also showed that the TST could easily accumulate Zn in the bodies of *P. viridis* when compared to Cd and Cu. This implied that the BYS could act as an effective biopolymer for monitoring Zn pollution during accumulation (or exposure) in polluted coastal waters.

The as-expected higher levels of metals in the bivalves transplanted to more polluted coastal waters are supported by several bivalve transplantation studies reported in the literature. For instance, those considering the TST of *M. galloprovincialis* [16,17,18,19,20,21,22,24,25] and *P. viridis* [23] in field-based transplantation studies. However, the reported studies did not analyze the BYS, making a comparison of the present findings rather impossible.

Yap [10] concluded that the BYS of *P. viridis* served as an excretion route for Cd, Pb, and Zn under controlled laboratory conditions during accumulation and depuration tests. This resulted in a low possibility of Cd, Pb, and Zn ion adsorption through the ambient seawater into the byssal threads. Yap [10] further demonstrated that the metals discovered in the *P. viridis* BYS were more likely due to the assimilation of soft mussel tissues than to metal absorption from surrounding seawater. The use of the mussel BYS as a reliable biopolymer monitoring organ for Cd, Cu, and Zn contamination was thus further confirmed by these findings. Using laboratory experiments, Yap [10] found that the BYS of *P. viridis* exhibited a pattern of metal accumulation and depuration that was identical to that of the fecal contents, with the exception that it was unable to reach metal levels comparable to those in the untreated control mussel BYS. Since certain proportions of Cd, Pb, and Zn were deposited and kept or fixed in the different tissues of *P. viridis*, this behavior was predicted, since not all metals were eliminated from the mussel tissues. Although the likelihood of metals clinging to the byssal thread surfaces could not be completely ruled out, the quick loss during the early depuration stage was due to the mussel BYS functioning as a pathway for waste metal excretion following engagement in the metabolic systems [9]. According to Yap [10], the concentrations of Cd, Pb, and Zn in the *P. viridis* BYS were likewise found to be greater than in the TST of the mussels. These high concentrations of Cd, Pb, and Zn in the BYS and feces showed that these metals were removed from the TST throughout both the accumulation and depuration processes. Due to the higher quantities of these metals in the BYS and feces, these bodily fluids were more susceptible to Cd and Zn throughout both the accumulation and depuration phases. Defecation was assumed to be one of the processes by which some of the Zn was quickly eliminated from the TST when it reached the cytoplasm of *P. viridis* [95,96]. As a result, Cd and Cu were depurated more slowly than Zn because they were likely stored in metallothionein or membrane-bound granules. The accumulation and depuration investigations provided more evidence of the importance of the BYS and TST in the biological control of Cd, Cu, and Zn [60].

Metallothionein is secreted to reduce metal toxicity at high metal concentrations [97]. Although its exact functions are still unknown, it is well-acknowledged that MT plays a role in regulating the concentrations of essential metals such as Cu and Zn for cell growth and development [98]. Contrarily, Cu and Zn may be regulated in bivalves and are acknowledged as crucial metals for metabolic activities [8,98].

In contrast to mytilids from the TST, which had the lowest levels of Cd, Szefer et al. [59] found that mytilids from more industrialized regions had substantially higher concentrations of Cu and Zn in their BYS. The BYS of mytilids was shown to be more selective and sensitive to fluctuations in metal concentrations in the environment compared to the TST, which suggested that the mussel BYS is an ideal biopolymer monitoring organ for detecting coastal regions vulnerable to harmful metal inputs.

### 4.4. The Role of Byssal Threads in Zn, Cu, and Cd Excretion

The similar patterns of the accumulation and elimination of Cd, Zn, and Cu in the BYS and TST during our field experimental study in the SOJ indicated the close relationship between the two entities, as was well-supported by the significant correlation coefficients based on the 34 populations. It can be assumed that the BYS generally acts as a Cd, Zn, and Cu excretion route. For instance, the significantly higher levels of Zn in the BYS than in the TST with increasing Zn accumulation indicated that the BYS acted as an excretion route during the accumulation period. The consistently and significantly higher levels in the BYS compared to the TST with decreasing Zn elimination indicated that the BYS acted as an excretion route during the depuration period. The *P. viridis* BYS and TST showed their value for tracking metal pollution in coastal waters. Our findings concurred with those of Yap et al. [8,60], who demonstrated that *P. viridis* was an effective accumulator of Cd, Cu, and Zn.

In addition, the differences in the number of influential factors selected between CdBYS and CdTST indicated that Cd differed from Zn and Cu, for which an equal number of influential factors were selected regarding their accumulation in both the BYS and TST. This also implied that Cd is a non-essential metal, while Zn and Cu are essential metals. In sum, it could be concluded that the strategies for the accumulation and regulation of Cd, Zn, and Cu in the BYS were clearly different from those in the TST of *P. viridis*. It is noteworthy that the BYS also acted as an effective excretion route of Cd, Zn, and Cu, regardless of their essentiality differences. 

According to Yap et al. [12], the BYS acts as a short-term compartment for Cd and Zn elimination. Heavy metals are reportedly excreted by *M. edulis* through the BYS [5,6,7]. Because of this, the higher Cu and Zn concentrations in the BYS than in the TST showed that the extra metals in the cellular cytosol were excreted as waste through the BYS.

The substantial metal accumulation found in the BYS may have been caused by the material used to build it. The byssal formation contains a large amount of hard-tanned protein [5]. Collagen, a protein released by a byssal gland in the foot, makes up the BYS, predominantly composed of the amino acids glycine and proline [8,82]. According to Coombs and Keller [5], the byssal threads are responsible for removing heavy metals from the nearby seawater. Several researchers [5,87,90,99] have suggested that the BYS significantly eliminates several trace elements, such as Fe and Zn, from the mussel’s body. Metal pollutants are transferred from the TST to the byssal threads rather than being adsorbed onto the surface of the BYS by the ambient environment (seawater, particulate matter, bottom sediments). According to Coombs and Keller [5], the *Mytilus* BYS may concentrate on different metals, sometimes to astonishing degrees, potentially through direct adsorption from the ambient fluids and/or by removal from the TST. This byssal substance contains the protein collagen, which has specific potential metal-binding sites predominantly made up of the amino acids glycine and proline. The BYS may therefore be a reliable gauge of how contaminated mollusks’ maritime environments are. The BYS extracts a sizeable amount of components from the mollusk’s body. Therefore, metallic contaminants (Fe, Zn, and As) are transferred from the TST to the BYS rather than being adsorbed onto its surface [5,99]. According to Coombs and Keller [5] and George et al. [90], the BYS is essential for eliminating Fe and Zn. The BYS matrix exhibits good metal ion absorption while considerably changing its surface structure, as Montroni et al. [3] claimed. They showed that polluted industrial and environmental fluids might have metal ions removed using the BYS, a waste product that causes environmental issues. The results of Holten-Andersen et al. [4] suggested that dopa–metal complexes may provide significant interactions for the integrity of composite cuticles distorted under strain.

### 4.5. The Prospects of Using the Byssus of Perna viridis as a Biomonitoring Biopolymer for Potentially Toxic Metals

The effective demonstration and subsequent use of the byssus of *P. viridis* as a good biomonitoring biopolymer for PTMs have constituted a long journey, involving hurdles and debates. The idea of using the mussel BYS first proposed by Prof. Dr. Ikuta from Miyazaki University of Japan in 1986 [6,7], followed by the extensive field-based collection of samples of many temperate mussels and oysters by Prof. Dr. Szefer from the Medical University of Gdansk of Poland between 1997 and 2006 [59,80,81,82,86,100,101,102] from different coastal waters worldwide have had a strong legacy in the ecotoxicological literature. They provided fundamental data-driven evidence that is, nevertheless, not 100% accepted. Their works contributed the most important scientific knowledge for determining better and more accurate biomonitoring tools to understand the ecotoxicology of potentially toxic and newly emerging chemicals.

The majority of the Sustainable Development Goals [103]—especially the objective related to “Good health and well-being”—can be met. The usage of the mussel BYS could be a catalyst and a crucial contributor to the current Nexus idea, if we consider the Nexus cycle (water–food–energy) [104]. It is believed that the application of the mussel BYS will play a significant role in developing the Nexus approach [104] to connect the integration of food, energy, and water in order to accommodate new concepts that are constantly expanding, such as a circular and green economy [105]. The concept of employing the mussel BYS, whether directly or indirectly, offers a potential future research direction to supplement the International Mussel Watch of the 1970s [106], which should be continued for a better environmental future [107,108,109,110,111].

## 5. Conclusions

In conclusion, the current study provided three significant pieces of evidence that support the use of BYS of *P. viridis* as a biomonitoring biopolymer for Zn, Cu, and Cd pollution in coastal waters. First, it was shown that the BYS was a more sensitive, concentrative, and accumulative biopolymer for the three metals than the TST according to the 34 field-collected populations with a ratio of BYS/TST > 1. The TST and byssal concentrations of the three metals showed highly significant relationships (*p* < 0.05). Second, the interspecific comparison between the current data and those previously published (for *Mytilidae* from Hong Kong, Japan, and Poland (Baltic Sea)) provided strong support for the data obtained in the current study. It showed that the *P. viridis* BYS was a significantly better biomonitoring biopolymer for identifying coastal areas exposed to Zn, followed by Cu and Cd, contamination, as well as its function as a pathway for the excretion of metal wastes. Third, the BYS was more reflective of the metal bioavailability and pollution of Zn in coastal waters, as evidenced by the greater correlation coefficients of metals between the BYS and sedimentary geochemical fractions than the TST and sedimentary geochemical fractions. Fourth, the most significant findings from the field-based experimental transplantation study showed unequivocally how the three metals were accumulated and eliminated in the BYS in both polluted and unpolluted areas in the SOJ. In conclusion, it was established that the *P. viridis* BYS is a superior biopolymer monitoring method compared to the TST for determining the bioavailability and contamination of Zn, Cu, and Cu in tropical coastal waters. Nonetheless, the accuracy of using the proposed mussel BYS for the monitoring of other PTMs still requires much research, considering the newly emerging chemical pollutants that must be detected in coastal areas, in addition to climate change factors and other unpredictable occurrences.

## Figures and Tables

**Table 1:** Overall statistics of concentrations (mg/kg dry weight) of Cd in the byssus (BYS) and total soft tissues (TST) of *Perna viridis* and their ratios (BYS/TST), collected from 34 populations in the coastal waters of Peninsular Malaysia (PM) and 2 sites in the Straits of Johore (SOJ) during experiment from the present study, along with those reported in the literature.

	SOJ (PV; N = 8)			GG (MET; N = 6)			PB (MET; N = 12)			HK (PV; N = 7)		
	TST	BYS	BYS/TST	TST	BYS	BYS/TST	TST	BYS	BYS/TST	TST	BYS	BYS/TST
Minimum	1.49	2.32	0.81	1.23	0.30	0.19	1.74	0.28	0.06	1.02	0.77	0.28
Maximum	4.09	6.06	2.25	3.12	1.20	0.63	6.07	3.25	0.74	5.40	1.83	1.16
Mean	2.72	3.52	1.38	1.92	0.81	0.42	3.09	1.00	0.32	3.01	1.34	0.60
Median	2.85	3.43	1.31	1.81	0.81	0.43	2.46	0.83	0.28	3.13	1.42	0.49
SD	0.88	1.16	0.48	0.65	0.35	0.15	1.34	0.82	0.18	1.87	0.36	0.34
SE	0.31	0.41	0.17	0.26	0.14	0.06	0.39	0.24	0.05	0.71	0.14	0.13
Skewness	0.08	1.30	0.58	1.08	−0.21	−0.21	1.03	1.90	0.98	0.04	−0.36	0.75
Kurtosis	−1.11	0.99	−0.46	0.16	−1.25	−0.81	−0.07	2.85	0.74	−1.71	−0.91	−0.93
	PM (PV; N = 34)			JP (ME; N = 4)			SB (MET; N = 5)			Korea (MG; N = 7)		
	TST	BYS	BYS/TST	TST	BYS	BYS/TST	TST	BYS	BYS/TST	TST	BYS	BYS/TST
Minimum	0.35	0.48	0.07	0.92	0.16	0.03	2.71	0.62	0.20	0.63	0.19	0.23
Maximum	7.04	6.06	4.55	18.4	1.05	0.88	5.57	3.65	1.07	9.98	5.40	1.89
Mean	2.04	2.35	1.81	5.51	0.56	0.36	3.52	1.92	0.55	3.17	1.69	0.71
Median	1.51	2.14	1.68	1.36	0.52	0.27	3.17	2.00	0.45	1.29	1.19	0.52
SD	1.88	1.13	1.11	8.60	0.38	0.39	1.18	1.25	0.35	3.49	1.73	0.57
SE	0.32	0.19	0.19	4.30	0.19	0.19	0.53	0.56	0.16	1.32	0.65	0.22
Skewness	1.96	0.96	0.66	1.15	0.33	0.58	1.28	0.26	0.55	1.23	1.60	1.42
Kurtosis	2.54	1.87	0.03	−0.67	−1.21	−1.24	−0.04	−1.29	−1.12	−0.01	1.24	0.67

Note: Cited from Hong Kong (HK) (KotO and Kennedy (K)) ([8] for *Perna viridis* (PV)); Korea (Masan and Ulsan) ([81] for *Mytilus galloprovincialis* (MG)); Pomeranian Bay (PB) ([59] for *M. edulis trossulus* (MET)); Supsk Bank (SB) ([59] for MET); Gulf of Gdansk (GG) ([59] for MET); and Japan (JP) ([6,82] for *M. edulis* (ME)).

**Table 2:** Overall statistics of concentrations (mg/kg dry weight) of Zn in the byssus (BYS) and total soft tissues (TST) of *Perna viridis* and their ratios (BYS/TST), collected from 34 populations in the coastal waters of Peninsular Malaysia (PM) and 2 sites in the Straits of Johore (SOJ) during experiment from the present study, along with those reported in the literature.

	SOJ (PV; N = 8)			GG (MET; N = 6)			PB (MET; N = 12)			HK (PV; N = 7)		
	TST	BYS	BYS/TST	TST	BYS	BYS/TST	TST	BYS	BYS/TST	TST	BYS	BYS/TST
Minimum	58.7	56.0	0.88	100	121	0.94	102	87.9	0.62	104	103	0.90
Maximum	144	185	2.05	151	226	1.77	193	396	2.37	152	342	2.71
Mean	90.7	122	1.35	126	163	1.30	159	227	1.40	119	202	1.65
Median	82.6	124	1.28	123	144	1.22	162	215	1.36	115	139	1.28
SD	29.8	48.9	0.41	17.7	43.8	0.30	23.6	87.8	0.43	16.4	110	0.80
SE	10.6	17.3	0.14	7.21	17.9	0.12	6.80	25.4	0.12	6.19	41.6	0.30
Skewness	0.71	−0.08	0.49	0.03	0.62	0.52	−0.99	0.36	0.54	1.34	0.30	0.48
Kurtosis	−0.75	−1.41	−0.89	−0.82	−1.38	−0.92	1.05	−0.54	0.76	0.57	−1.81	−1.50
	PM (PV; N = 34)			JP (ME; N = 4)			SB (MET; N = 5)			Korea (MG; N = 7)		
	TST	BYS	BYS/TST	TST	BYS	BYS/TST	TST	BYS	BYS/TST	TST	BYS	BYS/TST
Minimum	50.9	34.9	0.36	127	98.4	0.77	124	120	0.88	107	81.1	0.68
Maximum	130	256	2.91	360	297	1.00	157	264	2.12	279	536	3.00
Mean	95.0	126	1.35	241	211	0.87	140	170	1.23	165	331	2.01
Median	97.6	114	1.30	239	224	0.86	135	139	0.97	141	387	1.92
SD	23.0	61.8	0.62	95.3	83.9	0.10	16.8	61.1	0.52	65.3	157	0.83
SE	3.95	10.6	0.11	47.6	41.9	0.05	7.50	27.3	0.23	24.7	59.3	0.31
Skewness	−0.18	0.44	0.53	0.09	−0.48	0.47	0.21	0.78	1.23	0.93	−0.54	−0.31
Kurtosis	−1.17	−0.65	−0.14	−1.01	−1.07	−1.10	−1.77	−0.97	−0.17	−0.75	−0.86	−1.07

Note: Cited from Hong Kong (HK) (KotO and Kennedy (K)) ([8] for *Perna viridis* (PV)); Korea (Masan and Ulsan) ([81] for *Mytilus galloprovincialis* (MG)); Pomeranian Bay (PB) ([59] for *M. edulis trossulus* (MET)); Supsk Bank (SB) ([59] for MET); Gulf of Gdansk (GG) ([59] for MET); and Japan (JP) ([6,82] for *M. edulis* (ME)).

**Table 3:** Overall statistics of concentrations (mg/kg dry weight) of Cu in the byssus (BYS) and total soft tissues (TST) of *Perna viridis* and their ratios (BYS/TST), collected from 34 populations in the coastal waters of Peninsular Malaysia (PM) and 2 sites in the Straits of Johore (SOJ) during experiment from the present study, along with those reported in the literature.

	SOJ (PV; N = 8)			GG (MET; N = 6)			PB (MET; N = 12)			HK (PV; N = 7)		
	TST	BYS	BYS/TST	TST	BYS	BYS/TST	TST	BYS	BYS/TST	TST	BYS	BYS/TST
Minimum	10.0	8.55	0.55	5.70	17.3	2.40	6.63	17.9	2.37	10.1	32.6	2.38
Maximum	33.8	37.8	1.12	8.93	33.6	4.56	23.9	58.1	2.96	18.0	111	6.17
Mean	21.9	17.4	0.79	7.14	24.5	3.45	10.0	25.9	2.61	14.7	60.1	3.98
Median	23.1	14.4	0.79	7.26	24.0	3.42	8.83	23.5	2.57	15.8	44.8	3.44
SD	7.92	9.28	0.22	1.14	5.96	0.74	4.55	10.7	0.19	3.23	31.3	1.49
SE	2.80	3.28	0.08	0.47	2.43	0.30	1.31	3.08	0.05	1.22	11.8	0.56
Skewness	−0.15	1.43	0.37	0.28	0.35	0.12	2.67	2.49	0.58	−0.63	0.77	0.61
Kurtosis	−0.96	1.00	−1.22	−0.77	−1.04	−0.72	5.85	5.28	−0.69	−1.22	−1.07	−1.21
	PM (PV; N = 34)			JP (ME; N = 4)			SB (MET; N = 5)			Korea (MG; N = 7)		
	TST	BYS	BYS/TST	TST	BYS	BYS/TST	TST	BYS	BYS/TST	TST	BYS	BYS/TST
Minimum	2.82	8.55	0.83	3.76	18.8	3.52	7.62	11.0	1.40	6.99	21.9	2.74
Maximum	20.1	135.3	6.73	385	1870	16.9	10.3	32.6	3.17	58.8	211	6.31
Mean	10.4	34.8	3.26	112	697	7.57	8.38	21.3	2.50	17.2	73.0	4.51
Median	10.8	27.4	2.99	29.2	449	4.93	7.87	19.7	2.52	8.76	49.6	4.72
SD	3.64	24.7	1.45	183	879	6.26	1.09	7.94	0.68	18.8	66.8	1.35
SE	0.62	4.24	0.25	91.7	440	3.13	0.49	3.55	0.30	7.09	25.3	0.51
Skewness	0.46	2.09	0.43	1.11	0.57	1.11	1.29	0.20	−0.84	1.87	1.42	−0.05
Kurtosis	0.90	6.01	−0.47	−0.71	−1.28	−0.69	−0.06	−0.82	−0.51	1.74	0.68	−1.48

Note: Cited from Hong Kong (HK) (KotO and Kennedy (K)) ([8] for *Perna viridis* (PV)); Korea (Masan and Ulsan) ([81] for *Mytilus galloprovincialis* (MG)); Pomeranian Bay (PB) ([59] for *M. edulis trossulus* (MET)); Supsk Bank (SB) ([59] for MET); Gulf of Gdansk (GG) ([59] for MET); and Japan (JP) ([6,82] for *M. edulis* (ME)).

**Table 4:** Correlation coefficients of metals (Cd, Cu, and Zn) between byssus (BYS) and total soft tissue (TST) of *Perna viridis* and their geochemical fractions in the surface sediments collected from 34 populations in the coastal waters of Peninsular Malaysia. N = 34.

	ZnBYS	ZnTST	ZnF1	ZnF2	ZnF3	ZnF4	ZnSUM
ZnBYS	1.00	0.39	0.28	0.15	0.18	0.01	0.22
ZnTST	0.39	1.00	0.29	0.10	0.03	0.12	0.05
CuBYS	0.66	0.37	0.53	0.47	0.42	0.06	0.44
CuTST	0.36	0.45	0.63	0.48	0.42	0.07	0.43
CdBYS	0.13	0.05	0.20	0.09	−0.06	−0.15	−0.01
CdTST	−0.43	0.20	0.29	0.24	0.26	0.08	0.18
	CuBYS	CuTST	CuF1	CuF2	CuF3	CuF4	CuSUM
ZnBYS	0.66	0.36	0.21	−0.15	0.37	0.24	0.32
ZnTST	0.37	0.45	0.01	−0.09	0.29	0.11	0.19
CuBYS	1.00	0.69	0.49	−0.03	0.56	0.48	0.58
CuTST	0.69	1.00	0.57	0.08	0.51	0.34	0.50
CdBYS	0.03	0.18	0.10	0.08	0.04	−0.08	0.03
CdTST	0.07	0.17	0.07	0.12	0.14	0.02	0.10
	CdBYS	CdTST	CdF1	CdF2	CdF3	CdF4	CdSUM
ZnBYS	0.13	−0.43	0.29	0.49	0.34	0.15	0.43
ZnTST	0.05	0.20	0.17	0.01	0.13	−0.16	−0.03
CuBYS	0.03	0.07	0.28	0.30	0.30	0.19	0.34
CuTST	0.18	0.17	0.30	0.12	0.24	−0.02	0.15
CdBYS	1.00	−0.16	0.28	0.15	0.25	−0.19	0.07
CdTST	−0.16	1.00	0.08	−0.01	0.05	−0.16	−0.09

Note: Values in red are significantly correlated at <0.05; F1 = easily, freely leachable or exchangeable fraction; F2 = acid-reducible fraction; F3 = oxidizable organic fraction; F4 = resistant fraction; SUM = summation of F1, F2, F3, and F4.

**Table 5:** Multiple linear stepwise regression analytical outputs based on byssus (BYS) and total soft tissue (TST) of *Perna viridis* as dependent variables; independent variables are the geochemical fractions of the six metals collected from 34 populations in the coastal waters of Peninsular Malaysia.

ZnBYS	Intercept	CuBYS	CdTST	CdF2	ZnTST	CuF2	ZnF2	CdF3	CuF1			R	F	df
	−33.74	0.286	−0.56	0.75	0.359	−0.26	0.439	−0.24	−0.17			0.958	34.9	8.25
ZnTST	Intercept	CuTST	CuF1	CuF3	ZnSUM	ZnF4	CdF4	CdF3	CuF2			R	F	df
	205.8	0.537	−0.6	1.04	−0.91	0.653	−0.22	0.322	−0.18			0.814	6.12	8.25
CuBYS	Intercept	CuTST	ZnBYS	CdTST	CuF4	ZnTST	CdF2					R	F	df
	0.575	0.396	0.8	0.384	0.201	−0.22	−0.17					0.888	16.9	6.27
CuTST	Intercept	CuBYS	ZnF1	ZnTST	CdF3	CuF1	CuF3					R	F	df
	−3.3	0.415	0.368	0.359	−0.15	0.593	−0.53					0.845	11.25	6.27
CdBYS	Intercept	CdF1	CdF4	CdTST	CuF2							R	F	df
	0.522	0.375	−0.32	−0.27	0.201							0.473	2.09	4.29
CdTST	Intercept	ZnBYS	CuBYS	ZnTST	CdF2	CuF2	ZnF2	CdF3	CuF1	ZnF1	CdBYS	R	F	df
	0.086	−1.3	0.301	0.461	1.05	−0.21	0.628	−0.49	−0.47	0.376	−0.14	0.914	11.64	10.23

Note: The dependent variables were the metals in the TST and BYS of the mussels; the independent variables were the sedimentary geochemical fractions (F1, F1, F3, F4, and SUM). All values were log_10_ (value + 1)-transformed prior to multiple (forward) stepwise regression analysis. According to the Shapiro–Wilk test of normality, the R values were *p* < 0.05 for all the geochemical fractions of the six metals (i.e., the independent variables that were log10 transformed), except for ZnBYS, ZnTST, ZnF4, CuTST, CuF2, and CdF4. For all equations, N = 34.

## Data Availability

Not applicable.

## Supplementary Materials

The following supporting information can be downloaded at: https://www.mdpi.com/article/10.3390/biology12040523/s1, Table S1: Information on field sampling of Perna viridis collected between 1998 and 2008 used in the present study, Table S2: The measurements of shell length (mean ± SE, mm); shell width (mm); and shell eight (mm) during the field experimental study for Kg. Sg. Melayu to Kg. Pasir Puteh (M-P) and Kg. Pasir Puteh to Kg. Sg. Melayu (P-M), from W0 to W12, in the Straits of Johore, Table S3: A comparison of heavy metal concentrations (mg/kg dry weight) between measured values and certified values according to the Certified Reference Materials for dogfish liver (DOLT-3) and Chinese soil (NSC DC73319), Table S4: Statistical outputs of normality test using Shapiro–Wilk method on the data used for the correlation and regression analyses; p values < 0.05 (in bold) deviated from normality and were log10 (mean + 1)-transformed prior to statistical analysis in the present study, Table S5: Comparison of concentrations of Cd, Cu, and Zn in the geochemical fractions of the surface sediments between Kg. Pasir Puteh (KPP) and Kg. Sungai Melayu (KSM), Table S6: Concentrations (mg/kg dry weight) of Cd in the byssus (BYS) and total soft tissues (TST) of Perna viridis and their geochemical fractions in the surface sediments, collected from 34 populations in the coastal waters of Peninsular Malaysia, Table S7: Concentrations of Cd, Cu, and Zn in the total soft tissues and byssus of marine mussels cited from Hong Kong (KotO and Kennedy (K)) (Nicholson and Szefer (2003) for Perna viridis (PV)) (No. 1); Korea (Masan and Ulsan) (Szefer et al. (2004) for Mytilus galloprovincialis (MG)) (No. 2); Pomeranian Bay (PB) (Szefer et al. (2002) for Mytilus edulis trossulus (MET)) (No. 3); Supsk Bank (SB) (Szefer et al. (2002) for MET) (No. 3); Gulf of Gdansk (GG) (Szefer et al. (2002) for MET) (No. 3); and Japan (Ikuta, 1986a; Szefer at al., 1999) (No. 4,5) for Mytilus edulis (ME). TS = this study, Table S8: Concentrations (mg/kg dry weight) of Zn in the byssus (BYS) and total soft tissues (TST) of Perna viridis, and their geochemical fractions in the surface sediments collected from 34 populations in the coastal waters of Peninsular Malaysia, Table S9: Concentrations (mg/kg dry weight) of Cu in the byssus (BYS) and total soft tissues (TST) of Perna viridis and their geochemical fractions in the surface sediments collected from 34 populations in the coastal waters of Peninsular Malaysia.
