# Supplementary material for: Byssus of Green-Lipped Mussel Perna viridis as a Biomonitoring Biopolymer for Zinc Pollution in Coastal Waters
Source: Biology (Basel). 2023 Mar 30;12(4):523. doi: 10.3390/biology12040523 (PMC10136061; doi:10.3390/biology12040523)
Supplement: Supplementary file 1 [file biology-12-00523-s001.zip › biology-2267853-supplementary.pdf]

biology

Article

Byssus of Green-Lipped Mussel *Perna viridis* as a Biomonitoring Biopolymer for Zinc Pollution in Coastal Waters

Chee Kong Yap [1,*] and Khalid Awadh Al-Mutairi [2]

1 Department of Biology, Faculty of Science, Universiti Putra Malaysia, Serdang 43400 UPM, Selangor, Malaysia
2 Department of Biology, Faculty of Science, University of Tabuk, Tabuk P.O. Box 741, Saudi Arabia
* Correspondence: yapckong@hotmail.com or yapchee@upm.edu.my

Table S1: Information on field sampling of *Perna viridis* collected between 1998-2008 used in the present study.

PN	SN	Sampling sites	Year		Shell length (mm)
1	1	Bagan Lalang, Selangor	1998	Blalang1998	91.2 (79.2– 104.2)
2	2	Bagan Tiang, Perak	2005	Btiang2005	85.10
3	3	Gelang Patah, Johore	2004	Gpatah2004	40.0
4	4	Kuala Linggi, Negeri Sembilan	2000	Klinggi2000	80.0 (75.0– 98.6)
5	5	Kg. Masai, Johore	2004	Kmasai2004	45.0
6	6	Kuala Pontian, Pahang	2004	Kpontian2004	70.84
7			2007	Kpontian2007	41.0 (30-45)
8	7	Kg. Pasir Puteh, Johore	2000	KPPuteh2000	61.1 (51.8– 91.3)
9			2005	KPPuteh2005	65.0-84.0
10			2007	KPPuteh2007	56.1 (50-65)
11			2008	KPPuteh2008	48. 8 (45.9-50.8)
12			2009	KPPuteh2009	34.5
13	8	Kg. Sungai Ayam, Johore	2008	KSAyam2008	NA
14	9	Kg. Sungai Melayu, Johore	2009	KSMelayu2009	30.5
15	10	Kukup, Johore	2005	Kukup2005	83.0
16			2007	Kukup2007	43.8 (35-50)
17	11	Lukut, Negeri Sembilan	1998	Lukut1998	93.9 (82.2– 107.2)
18	12	Minyak Beku, Malacca	2005	Mbeku2005	76.0
19	13	Nenasi, Pahang	2004	Nenasi2004	76.1
20	14	Pulau Aman, Penang	1999	Paman1999	91.5 (72.0– 112.5)
21	15	Pantai Lido, Johore	1998	PLido1998	59.4 (46.8– 72.2)
22			2004	Plido2004	63.0
23			2005	PLido2005	97.0
24			2007	Plido2007	82.0 (65-105)
25			2008	PLido2008	NA
26	16	Pasir Panjang, Negeri Sembilan	2000	Ppanjang2000 {1998}	88.6 (68.1– 109.1)
27	17	Sebatu, Malacca	2000	Sebatu2000	85.4 (75.2– 88.0)
28			2004	Sebatu2004	75.5

29	18	Senibong, Johore	2004	Senibong2004	62.0
30			2007	Senibong2007	38.4 (35-45)
31			2008	Senibong2008	69.7
32	19	Telok Jawa, Johore	2004	Tjawa2004	84.0
33	20	Tanjung Kupang, Johore	2000	Tkupang2000	83.6 (70.0– 90.0)
34			2007	Tkupang2007	48.0 (40-55)

Note: PN= Population number; S= Geographical sampling site; NA= not available.

Table S2: The measurements of shell length (mean ± SE, mm), shell width (mm) and shell eight (mm), during the field experimental study from Kg. Sg. Melayu to Kg. Pasir Puteh (M-P), and from Kg. Pasir Puteh to Kg. Sg. Melayu (P-M), from week (W) 0 to W12, at the Straits of Johore.

M-P	Shell Length			Shell width			Shell height		
W0	30.45	±	2.64	9.07	±	1.04	14.64	±	1.54
W2	56.67	±	3.68	16.77	±	3.67	23.94	±	3.19
W6	68.88	±	4.51	20.67	±	3.92	29.22	±	2.06
W10	79.94	±	6.37	24.38	±	2.83	35.17	±	3.17
P-M	Shell Length			Shell width			Shell height		
0	34.46	±	1.33	10.22	±	1.26	15.66	±	2.37
2	53.17	±	3.14	16.96	±	3.11	22.39	±	3.88
6	66.15	±	4.57	19.37	±	4.31	29.61	±	4.32
10	78.74	±	5.39	23.41	±	3.94	35.10	±	4.16

Table S3: A comparison of heavy metal concentrations (mg/kg dry weight) between measured values and certified values in the Certified Reference Materials for dogfish liver DOLT-3, and for Soil China (NSC DC73319).

Metals	Metals	Certified values (C)			Measured value (M)			Percentage of recovery [(M/[illegible] 100]
Cu	DOLT-3	31.20	±	1.00	26.81	±	0.25	85.93
	NSC	21.00	±	2.00	18.28	±	0.74	87.05
Cd	DOLT-3	19.40	±	0.60	14.68	±	0.34	75.67
	NSC	4.30	±	0.40	4.64	±	0.14	107.91
Zn	DOLT-3	86.60	±	2.40	76.12	±	0.81	99.90
	NSC	680.00	±	25.00	566.76	±	6.39	83.35

Table S4: Statistical outputs of normality test using Shapiro-Wilk tests on the data used for the correlation and regression analyses. P values < 0.05 (in bold) deviated from normality and were log10 (mean +1) transformed prior to statistical analysis in the present study. N=34.

	Statistics	df	P
ZnBYS	0.944	34	0.079
ZnTST	0.951	34	0.132
ZnF1	0.607	34	**0.000**
ZnF2	0.814	34	**0.000**
ZnF3	0.925	34	**0.022**
ZnF4	0.948	34	0.108
ZnSUM	0.889	34	**0.002**
CuBYS	0.809	34	**0.000**
CuTST	0.943	34	0.074
CuF1	0.840	34	**0.000**
CuF2	0.961	34	0.258
CuF3	0.743	34	**0.000**
CuF4	0.847	34	**0.000**
CuSUM	0.759	34	**0.000**
CdBYS	0.934	34	**0.041**
CdTST	0.666	34	**0.000**
CdF1	0.847	34	**0.000**
CdF2	0.592	34	**0.000**
CdF3	0.823	34	**0.000**
CdF4	0.952	34	0.138
CdSUM	0.889	34	**0.002**

Note: F1= easily, freely or exchangeable fraction; F2= acid-reducible fraction; F3= oxidisable-organic fraction; F4= resistant fraction; SUM= summation of F1, F2, F3 and F4.

Table S5: Comparison of concentrations of Cd, Cu and Zn in the geochemical fractions of the surface sediments between Kg. Pasir Puteh (KPP) and Kg. Sungai Melayu (KSM).

	L	Cd		Cu		Zn	
Week	Site	KPP	KSM	KPP	KSM	KPP	KSM
0	F1	0.68	0.23	1.07	0.42	20.56	2.26
	F2	0.72	0.57	0.18	0.33	78.90	2.12
	F3	0.84	0.50	71.20	9.67	109.07	58.78
	F4	0.16	0.13	15.49	6.62	35.32	15.73
	SUM	2.40	1.44	87.93	17.05	243.86	78.89
6	F1	0.73	0.17	2.20	0.99	23.08	2.66
	F2	0.63	0.50	0.40	0.47	84.63	2.18
	F3	0.99	0.47	91.87	20.24	116.00	55.15
	F4	0.28	0.33	19.79	12.19	51.73	15.38
	SUM	2.64	1.47	114.26	33.89	275.44	75.36

Note: F1= easily, freely or exchangeable fraction; F2= acid-reducible fraction; F3= oxidisable-organic fraction; F4= resistant fraction; SUM= summation of F1, F2, F3 and F4.

Table S6: Concentrations (mg/kg dry weight) of Cd in the byssus (BYS) and total soft tissues (TST) of *Perna viridis*, and their geochemical fractions in the surface sediments, collected from 34 populations on the coastal waters of Peninsular Malaysia.

	Cd	BYS	TST	F1	F2	F3	F4	SUM
1	Blalang1998	1.61	1.22	0.07	0.16	0.10	0.18	0.51
2	Btiang2005	1.70	1.97	0.16	0.21	0.20	1.13	1.69
3	Gpatah2004	2.46	2.04	0.16	1.65	0.23	1.28	3.33
4	Klinggi2000	1.59	1.21	0.41	0.18	0.36	1.59	2.54
5	Kmasai2004	1.93	1.67	0.23	0.59	0.23	1.79	2.84
6	Kpontian2004	2.86	1.96	0.09	0.17	0.12	0.52	0.91
7	Kpontian2007	1.92	1.62	0.00	0.03	0.08	0.30	0.42
8	KPPuteh2000	2.55	0.82	0.22	0.19	0.33	0.45	1.24
9	KPPuteh2005	1.79	2.71	0.29	0.80	0.70	1.37	3.15
10	KPPuteh2007	2.68	1.80	0.24	0.46	0.32	0.32	1.34
11	KPPuteh2008	1.60	6.75	0.23	0.04	0.21	0.71	1.18
12	KPPuteh2009	6.06	2.61	0.68	0.72	0.84	0.16	2.40
13	KSAyam2008	0.48	7.04	0.18	0.10	0.11	0.76	1.14
14	KSMelayu2009	3.36	1.57	0.23	0.57	0.50	0.13	1.44
15	Kukup2005	3.95	2.22	0.14	0.09	0.07	0.93	1.23
16	Kukup2007	4.24	1.44	0.16	0.12	0.11	1.06	1.46
17	Lukut1998	1.75	0.76	0.04	0.14	0.19	0.47	0.84
18	Mbeku2005	1.99	1.11	0.20	0.12	0.15	1.86	2.33
19	Nenasi2004	2.25	2.23	0.19	0.26	0.23	1.02	1.71
20	Paman1999	0.87	0.87	0.27	0.11	0.02	0.88	1.29
21	PLido1998	3.10	0.68	0.12	0.10	0.06	0.62	1.05
22	Plido2004	2.19	1.08	0.10	2.24	0.65	1.20	4.19
23	PLido2005	2.19	1.08	0.30	0.47	0.28	1.16	2.21
24	Plido2007	1.67	0.98	0.04	0.18	0.21	0.80	1.23
25	PLido2008	0.49	6.91	0.12	0.12	0.10	0.64	0.99
26	Ppanjang2000	3.46	1.08	0.23	0.14	0.09	0.58	1.04
27	Sebatu2000	3.44	0.81	0.29	0.06	0.03	1.22	1.59
28	Sebatu2004	0.77	0.35	0.14	0.13	0.10	1.22	1.60
29	Senibong2004	2.09	1.17	0.22	0.25	0.46	2.31	3.23
30	Senibong2007	2.96	1.79	0.15	0.10	0.19	0.76	1.20
31	Senibong2008	3.36	6.77	0.10	0.13	0.11	0.49	0.84
32	Tjawa2004	2.06	1.69	0.22	0.46	0.31	1.92	2.90
33	Tkupang2000	1.75	0.51	0.14	0.12	0.15	0.66	1.07

34	Tkupang2007	2.79	0.83	0.08	0.07	0.11	0.74	1.00

Note: F1= easily, freely, leachable or exchangeable fraction; F2= acid-reducible fraction; F3= oxidisable-organic fraction; F4= resistant fraction; SUM= summation of F1, F2, F3 and F4.

Table S7: Concentrations of Cd, Cu and Zn in the total soft tissues and byssus of marine mussels cited from Hong Kong (KotO and Kennedy (K)) (Nicholson and Szefer (2003) for *Perna viridis* (PV) (No. 1)), Korea (Masan and Ulsan) (Szefer et al. (2004) for *Mytilus galloprovincialis* (MG) (No. 2), Pomeranian Bay (PB) (Szefer et al. (2002) for *Mytilus edulis trossulus* (MET)) (No. 3), Supsk Bank (SB) (Szefer et al. (2002) for MET) (No. 3), Gulf of Gdansk (GG) (Szefer et al. (2002) for MET) (No. 3), and Japan (Ikuta, 1986a; Szefer at al., 1999) (No. 4,5) for *Mytilus edulis* (ME). TS= This study.

		Zn			Cu			Cd			Reference
Sp.	Sites	ZnTST	ZnBYS	ZnBYS/ ZnTST	CuTST	CuBYS	CuBYS/ CuTST	CdTST	CdBYS	CdBYS/ CdTST	
PV	KatO-1	104	103	0.99	10.1	40.4	4.00	3.13	1.53	0.49	1
PV	KatO-2	115	104	0.90	10.4	32.6	3.13	4.59	1.42	0.31	1
PV	KatO-3	109	139	1.28	15.8	44.8	2.84	4.54	1.83	0.40	1
PV	KatO-4	108	112	1.04	14.9	35.4	2.38	5.40	1.53	0.28	1
PV	K-1	116	314	2.71	17.5	60.2	3.44	1.30	0.77	0.59	1
PV	K-2	126	342	2.71	18.0	111	6.17	1.02	0.97	0.95	1
PV	K-3	152	297	1.95	16.4	96.3	5.87	1.12	1.30	1.16	1
MG	Masan-1	124	372	3.00	8.76	47.6	5.43	0.63	1.19	1.89	2
MG	Masan-2	107	156	1.46	6.99	22.4	3.20	0.84	0.19	0.23	2
MG	Masan-3	119	81.1	0.68	7.99	21.9	2.74	1.29	0.67	0.52	2
MG	Ulsan-1	233	387	1.66	58.8	211	3.59	9.98	5.40	0.54	2
MG	Ulsan-2	279	536	1.92	18.9	105	5.56	5.86	2.04	0.35	2
MG	Ulsan-3	151	393	2.60	8.53	53.8	6.31	2.34	1.06	0.45	2
MG	Ulsan-4	141	392	2.78	10.5	49.6	4.72	1.28	1.27	0.99	2
MET	PB-1	172	260	1.51	9.06	26.8	2.96	2.25	0.94	0.42	3
MET	PB-2	143	87.9	0.62	8.61	22.1	2.57	1.74	0.34	0.20	3
MET	PB-3	181	340	1.88	23.9	58.1	2.42	2.16	0.49	0.23	3
MET	PB-4	151	153	1.02	7.46	18.4	2.43	2.15	0.61	0.28	3
MET	PB-5	168	223	1.33	9.60	25.2	2.62	2.66	0.78	0.29	3
MET	PB-6	158	197	1.25	9.78	24.9	2.55	4.69	0.28	0.06	3
MET	PB-7	102	138	1.36	6.63	17.9	2.70	2.14	0.88	0.41	3
MET	PB-8	167	396	2.37	8.34	24.1	2.89	3.21	0.87	0.27	3
MET	PB-9	152	206	1.35	8.31	22.9	2.76	4.37	3.25	0.74	3
MET	PB-10	193	275	1.42	11.2	28.7	2.57	6.07	1.14	0.19	3
MET	PB-11	176	277	1.58	9.04	22.6	2.50	3.58	1.83	0.51	3
MET	PB-12	145	168	1.16	8.00	18.9	2.37	2.06	0.56	0.27	3
MET	SB-1	157	139	0.88	10.3	32.6	3.17	5.57	2.51	0.45	3
MET	SB-2	157	198	1.26	7.87	11.0	1.40	2.71	0.81	0.30	3
MET	SB-3	124	120	0.97	7.62	18.8	2.47	2.75	2.00	0.73	3

MET	SB-4	135	127	0.94	8.34	24.4	2.93	3.17	0.62	0.20	3
MET	SB-5	125	264	2.12	7.80	19.7	2.52	3.40	3.65	1.07	3
MET	GG-1	100	121	1.21	5.70	17.3	3.03	1.55	0.30	0.19	3
MET	GG-2	119	134	1.13	7.14	27.6	3.86	1.23	0.58	0.47	3
MET	GG-3	128	226	1.77	7.38	33.6	4.56	1.74	0.91	0.52	3
MET	GG-4	151	143	0.94	8.93	21.4	2.40	3.12	1.20	0.38	3
MET	GG-5	119	145	1.22	6.14	20.3	3.30	1.88	1.18	0.63	3
MET	GG-6	138	211	1.53	7.53	26.7	3.54	2.01	0.71	0.35	3
ME	Urashiro	127	98.4	0.77	6.50	22.9	3.52	0.92	0.40	0.43	4
ME	Akamizu	244	243	1.00	51.8	876	16.91	1.52	0.16	0.11	4
ME	Saganoseki	360	297	0.83	385	1870	4.86	18.40	0.64	0.03	4
ME	Beppu	233	204	0.88	3.76	18.8	5.00	1.20	1.05	0.88	5
PV	P to M W0	85.8	176	2.05	33.8	37.8	1.12	4.09	6.06	1.48	TS
PV	P to M W2	79.3	137	1.72	28.8	22.8	0.79	3.5	2.84	0.81	TS
PV	P to M W6	71.1	111	1.56	22.5	17.4	0.78	2.99	2.53	0.85	TS
PV	P to M W10	58.7	64.4	1.10	12.9	13.8	1.07	1.92	2.32	1.21	TS
PV	M to P W0	63.5	56.0	0.88	10.0	8.55	0.86	1.49	3.36	2.25	TS
PV	M to P W2	101	92.9	0.92	18.6	11.0	0.59	1.96	3.49	1.78	TS
PV	M to P W6	121.8	154	1.27	23.6	13.0	0.55	2.71	3.74	1.38	TS
PV	M to P W10	144	184.7	1.28	25.6	15.0	0.59	3.08	3.81	1.24	TS
PV	Blalang1998	96.4	179.42	1.86	8.20	28.6	3.49	1.22	1.61	1.32	TS
PV	Btiang2005	85.9	122.42	1.42	11.4	22.2	1.94	1.97	1.70	0.86	TS
PV	Gpatah2004	59.4	172.74	2.91	9.09	37.1	4.08	2.04	2.46	1.21	TS
PV	Klinggi2000	101.1	164.95	1.63	9.14	32.4	3.54	1.21	1.59	1.31	TS
PV	Kmasai2004	72.7	193.17	2.66	11.4	50.2	4.39	1.67	1.93	1.15	TS
PV	Kpontian2004	127	94.92	0.75	10.3	22.2	2.15	1.96	2.86	1.46	TS
PV	Kpontian2007	82.2	65.04	0.79	2.82	11.6	4.12	1.62	1.92	1.18	TS
PV	KPPuteh2000	129	243	1.88	20.1	135	6.73	0.82	2.55	3.11	TS
PV	KPPuteh2005	130	104	0.80	11.1	23.7	2.14	2.71	1.79	0.66	TS
PV	KPPuteh2007	104	180	1.73	13.1	51.4	3.91	1.80	2.68	1.49	TS
PV	KPPuteh2008	119	65.2	0.55	12.2	48.8	4.01	6.75	1.60	0.24	TS
PV	KPPuteh2009	103	176	1.71	13.4	37.8	2.83	2.61	6.06	2.32	TS
PV	KSAyam2008	80.3	57.8	0.72	7.59	41.7	5.50	7.04	0.48	0.07	TS
PV	KSMelayu2009	67.2	56.0	0.83	8.74	8.55	0.98	1.57	3.36	2.13	TS
PV	Kukup2005	116	106	0.92	10.5	16.2	1.55	2.22	3.95	1.78	TS
PV	Kukup2007	51.0	34.9	0.68	6.58	12.9	1.97	1.44	4.24	2.94	TS
PV	Lukut1998	69.4	100	1.45	10.2	29.5	2.88	0.76	1.75	2.30	TS
PV	Mbeku2005	125	162	1.29	8.03	23.7	2.95	1.11	1.99	1.80	TS
PV	Nenasi2004	82.8	43.1	0.52	3.73	18.3	4.90	2.23	2.25	1.01	TS
PV	Paman1999	110	142	1.30	10.8	32.6	3.02	0.87	0.87	1.00	TS
PV	PLido1998	117	165	1.41	9.39	25.5	2.71	0.68	3.10	4.55	TS

PV	Plido2004	118	256	2.16	11.9	61.7	5.20	1.08	2.19	2.04	TS
PV	PLido2005	118	256	2.16	11.9	61.7	5.20	1.08	2.19	2.04	TS
PV	Plido2007	72.9	87.4	1.20	5.98	13.9	2.32	0.98	1.67	1.70	TS
PV	PLido2008	110	39.4	0.36	11.1	9.21	0.83	6.91	0.49	0.07	TS
PV	Ppanjang2000	98.9	138	1.39	10.9	24.2	2.22	1.08	3.46	3.20	TS
PV	Sebatu2000	75.1	96.1	1.28	11.2	18.6	1.66	0.81	3.44	4.25	TS
PV	Sebatu2004	63.2	81.9	1.30	11.4	18.9	1.66	0.35	0.77	2.22	TS
PV	Senibong2004	58.20	95.1	1.63	11.6	43.4	3.75	1.17	2.09	1.79	TS
PV	Senibong2007	120	124	1.04	18.0	50.9	2.83	1.79	2.96	1.65	TS
PV	Senibong2008	107	58.8	0.55	17.7	62.2	3.52	6.77	3.36	0.50	TS
PV	Tjawa2004	94.6	179	1.89	12.2	71.6	5.87	1.69	2.06	1.22	TS
PV	Tkupang2000	88.6	175	1.97	6.31	26.2	4.15	0.51	1.75	3.43	TS
PV	Tkupang2007	79.0	85.3	1.08	5.90	10.4	1.77	0.83	2.79	3.38	TS

Table S8: Concentrations (mg/kg dry weight) of Zn in the byssus (BYS) and total soft tissues (TST) of *Perna viridis*, and their geochemical fractions in the surface sediments, collected from 34 populations on the coastal waters of Peninsular Malaysia.

No.	Zn	BYS	TST	F1	F2	F3	F4	SUM
1	Blalang1998	179.42	96.36	1.19	6.79	22.98	37.91	68.87
2	Btiang2005	122.42	85.93	1.80	4.33	49.26	83.06	138.45
3	Gpatah2004	172.74	59.37	0.45	0.91	18.20	44.40	64.00
4	Klinggi2000	164.95	101.11	0.80	19.19	28.53	48.55	97.07
5	Kmasai2004	193.17	72.69	4.44	41.60	85.90	69.70	202.00
6	Kpontian2004	94.92	126.62	0.20	1.96	7.53	47.39	57.08
7	Kpontian2007	65.04	82.18	0.00	3.85	9.46	38.08	51.38
8	KPPuteh2000	242.82	128.90	6.91	49.83	91.72	95.48	243.90
9	KPPuteh2005	104.11	129.58	6.81	20.78	128.26	111.61	267.47
10	KPPuteh2007	180.09	103.98	0.00	20.12	40.10	53.91	114.13
11	KPPuteh2008	65.20	118.54	4.02	12.92	58.45	53.84	98.56
12	KPPuteh2009	176.24	103.36	20.56	78.90	109.07	35.32	243.86
13	KSAyam2008	57.78	80.34	0.94	13.03	56.97	75.47	150.32
14	KSMelayu2009	56.00	67.19	2.26	2.12	58.78	15.73	78.89
15	Kukup2005	106.24	115.63	0.51	6.20	6.79	57.60	71.10
16	Kukup2007	34.86	50.99	0.00	16.85	33.98	97.76	148.60
17	Lukut1998	100.47	69.40	2.00	21.37	50.77	17.21	91.38
18	Mbeku2005	161.98	125.29	0.68	4.55	6.62	85.32	97.17
19	Nenasi2004	43.13	82.75	0.13	0.93	7.86	48.35	57.26
20	Paman1999	142.41	109.60	0.29	3.70	35.80	46.49	86.30
21	PLido1998	164.69	116.90	0.75	1.45	18.74	7.86	28.80

22	Plido2004	256.14	118.49	1.22	1.81	83.40	36.02	122.44
23	PLido2005	256.14	118.49	4.10	20.40	16.77	42.14	83.41
24	Plido2007	87.37	72.96	1.51	21.75	43.18	36.49	102.92
25	PLido2008	39.36	110.28	1.45	15.02	55.94	47.76	99.08
26	Ppanjang2000	137.70	98.92	1.03	7.01	31.81	77.10	116.90
27	Sebatu2000	96.13	75.14	0.39	1.33	9.07	49.51	60.00
28	Sebatu2004	81.92	63.18	0.16	1.90	7.74	69.79	79.58
29	Senibong2004	95.06	58.20	3.81	48.30	83.00	32.20	167.00
30	Senibong2007	123.95	119.74	4.67	31.00	39.24	36.63	111.55
31	Senibong2008	58.79	107.16	5.89	45.93	59.96	31.71	104.10
32	Tjawa2004	179.03	94.63	4.06	42.90	85.20	38.00	170.00
33	Tkupang2000	174.56	88.58	0.89	9.74	54.63	61.40	126.70
34	Tkupang2007	85.29	79.02	0.00	11.35	27.07	37.45	75.87

Note: F1= easily, freely, leachable or exchangeable fraction; F2= acid-reducible fraction; F3= oxidisable-organic fraction; F4= resistant fraction; SUM= summation of F1, F2, F3 and F4.

Table S9: Concentrations (mg/kg dry weight) of Cu in the byssus (BYS) and total soft tissues (TST) of *Perna viridis*, and their geochemical fractions in the surface sediments, collected from 34 populations on the coastal waters of Peninsular Malaysia.

	Cu	BYS	TST	F1	F2	F3	F4	SUM
1	Blalang1998	28.65	8.20	0.14	0.03	8.59	3.14	11.90
2	Btiang2005	22.22	11.44	0.26	0.19	5.86	14.30	20.60
3	Gpatah2004	37.05	9.09	0.39	0.46	3.66	13.20	17.80
4	Klinggi2000	32.36	9.14	0.45	0.14	3.92	16.78	21.29
5	Kmasai2004	50.15	11.43	1.80	0.43	118.00	64.10	184.00
6	Kpontian2004	22.20	10.31	0.12	0.33	2.15	9.40	12.00
7	Kpontian2007	11.63	2.82	0.08	0.38	1.50	5.50	7.46
8	KPPuteh2000	135.26	20.10	1.59	0.33	90.01	48.26	140.20
9	KPPuteh2005	23.71	11.06	1.14	0.80	118.84	66.14	186.92
10	KPPuteh2007	51.41	13.13	0.29	0.53	13.71	28.20	42.73
11	KPPuteh2008	48.80	12.18	0.39	0.22	20.71	36.56	57.88
12	KPPuteh2009	37.81	13.36	1.07	0.18	71.20	15.49	87.93
13	KSAyam2008	41.71	7.59	0.16	0.10	6.87	28.15	35.27
14	KSMelayu2009	8.55	8.74	0.42	0.33	9.67	6.62	17.05
15	Kukup2005	16.20	10.45	0.03	0.11	10.72	17.74	28.60
16	Kukup2007	12.98	6.58	0.17	0.51	1.01	12.18	13.87
17	Lukut1998	29.47	10.22	1.16	0.09	38.35	32.88	72.47
18	Mbeku2005	23.70	8.03	0.19	0.05	22.81	22.76	45.80
19	Nenasi2004	18.27	3.73	0.08	0.40	2.85	11.77	15.11

20	Paman1999	32.64	10.80	0.17	0.04	6.39	8.90	15.51
21	PLido1998	25.49	9.39	0.26	0.15	5.70	9.15	15.26
22	Plido2004	61.65	11.86	0.75	0.44	30.80	25.90	57.89
23	PLido2005	61.65	11.87	0.39	0.25	29.41	17.61	47.66
24	Plido2007	13.90	5.98	1.30	0.63	69.64	52.36	123.93
25	PLido2008	9.21	11.09	0.61	0.35	11.12	10.66	22.74
26	Ppanjang2000	24.16	10.87	0.34	0.13	2.40	13.95	16.82
27	Sebatu2000	18.56	11.16	0.44	0.02	0.82	12.79	14.07
28	Sebatu2004	18.92	11.43	0.15	0.36	0.84	10.16	11.51
29	Senibong2004	43.36	11.55	1.78	0.44	132.00	53.30	187.00
30	Senibong2007	50.97	18.04	0.99	0.55	69.54	51.75	122.83
31	Senibong2008	62.22	17.66	1.37	0.32	28.20	7.24	37.13
32	Tjawa2004	71.64	12.20	0.88	0.26	94.80	48.40	144.00
33	Tkupang2000	26.19	6.31	0.23	0.01	6.69	20.93	27.86
34	Tkupang2007	10.43	5.90	0.15	0.40	3.88	13.80	18.22

+Note: F1= easily, freely, leachable or exchangeable fraction; F2= acid-reducible fraction; F3= oxidisable-organic fraction; F4= resistant fraction; SUM= summation of F1, F2, F3 and F4.
